# Modeling of Creep Behavior of Particulate Composites with Focus on Interfacial Adhesion Effect

**DOI:** 10.3390/ijms232214120

**Published:** 2022-11-15

**Authors:** Julian Rech, Esther Ramakers-van Dorp, Bernhard Möginger, Berenika Hausnerova

**Affiliations:** 1 Department of Natural Sciences, Bonn-Rhein-Sieg University of Applied Sciences, von-Liebig-Straße 20, 53359 Rheinbach, Germany; 2 Centre of Polymer Systems, University Institute, Tomas Bata University in Zlin, nam. T.G. Masaryka 5555, 76001 Zlin, Czech Republic; 3 Faculty of Technology, Tomas Bata University in Zlin, Vavreckova 275, 76001 Zlin, Czech Republic

**Keywords:** modeling, creep, creep compliance, particulate composite, adhesion factor, cube in cube model, elementary volume

## Abstract

Evaluation of creep compliance of particulate composites using empirical models always provides parameters depending on initial stress and material composition. The effort spent to connect model parameters with physical properties has not resulted in success yet. Further, during the creep, delamination between matrix and filler may occur depending on time and initial stress, reducing an interface adhesion and load transfer to filler particles. In this paper, the creep compliance curves of glass beads reinforced poly(butylene terephthalate) composites were fitted with Burgers and Findley models providing different sets of time-dependent model parameters for each initial stress. Despite the finding that the Findley model performs well in a primary creep, the Burgers model is more suitable if secondary creep comes into play; they allow only for a qualitative prediction of creep behavior because the interface adhesion and its time dependency is an implicit, hidden parameter. As Young’s modulus is a parameter of these models (and the majority of other creep models), it was selected to be introduced as a filler content-dependent parameter with the help of the *cube in cube* elementary volume approach of Paul. The analysis led to the time-dependent creep compliance that depends only on the time-dependent creep of the matrix and the normalized particle distance (or the filler volume content), and it allowed accounting for the adhesion effect. Comparison with the experimental data confirmed that the elementary volume-based creep compliance function can be used to predict the realistic creep behavior of particulate composites.

## 1. Introduction

The majority of lightweight design parts are realized using reinforced composites [1]. Knowledge of their reliable and relevant physico-mechanical materials data, such as stiffness, strength, or creep compliance, is indispensable for engineering and design purposes. Due to the viscoelastic nature of polymer matrices, the dimensional stability in structural applications, and the corresponding long-term behavior, are often estimated by either extrapolation of short-term data or under accelerated testing conditions using higher initial stresses, elevated temperatures, and relative humidity [2]. However, these investigations are time and energy-consuming, especially in the case of creep experiments. To overcome this, modeling as an analytical approach is currently used to predict the performance of the composites.

The creep behavior of composites depends on the creep response of a matrix and a dispersed phase (fibers or particles), the content of the dispersed phase, interface adhesion between filler and matrix, and experimental parameters such as temperature and initial stress [3]. During the creep experiment, the interface adhesion in the composites can be reduced (by, e.g., delamination), leading to accelerated creep. The creep compliances increase faster with time than in the case of pure viscoelastic creep. If the load transfer from matrix to filler is decreased, the creep behavior of composites is determined dominantly by the matrix creep.

The filler/matrix adhesion depends on the compatibility between the filler and the matrix. Bek et al. [4] compared the long-term creep compliances of neat and recycled polypropylene (PP) reinforced with untreated wood fibers. They found a superior creep resistance for neat PP composites and attributed it to a higher crystallinity and better interfacial adhesion due to trans-crystalline structures around the wood fibers occurring only for neat PP. Chandekar et al. [5] studied the effect of various chemical pre-treatments of jute fibers on the creep recovery behavior of PP-jute composites. The creep evaluation using the Burgers model revealed that creep strains decreased due to the chosen fiber pre-treatment in the following manner: untreated > potassium permanganate > alkali (NaOH) > silane > NaOH with maleic anhydride grafted PP. Similar results were presented by Marcovich and Villar [6] for the creep behavior of low-density polyethylene compounded with untreated wood flour and modified with both organic peroxide and MAH. They concluded that MAH increased the interfacial adhesion between filler and matrix. Furthermore, they found that the functionalization using organic peroxide hindered crystallization and led to composites with lower crystallinity and, thus, more pronounced creep behavior.

In general, the creep at constant load is divided into three ranges of deformation [7]. After an instantaneous elastic deformation due to applied initial stress, primary creep, characterized by a decreasing creep rate, occurs. After a certain time, the creep rate becomes almost constant, indicating the range of secondary creep. Finally, tertiary creep occurs with a fast-increasing creep rate leading to a fracture. To quantitatively describe the measured creep behavior of polymers and composites, several models were developed, Table 1. They can be divided into two categories [8]:

### 1.1. Rheological Models

They combine springs and dashpots in a suitable manner to reproduce measured behavior (Maxwell model, Kelvin/Voigt model, Burgers model, etc.). The model parameters are the stiffnesses of springs and viscosities of dashpots, which can partly be determined by experiments or fitting procedures.

### 1.2. Empirical Equations

They approximate the measured behavior and the model parameter are determined by fitting procedures.

The Burgers model is an *in-series* arrangement of the Maxwell model (elastic spring *E*_1_ in series with a viscous dashpot *η*_1_) and the Kelvin model (elastic spring *E*_2_ parallel to viscous dashpot *η*_2_) [9]. For the constant initial stress *σ*_0_, it reproduces qualitatively the creep behavior with the initial elastic strain and primary and secondary creep strains; Equation (1), Table 1. For long times, only the dashpot having viscosity *η*_2_ determines the creep behavior in a time-linear manner, which does not display the real creep behavior of most materials. To give the Burgers model more generality and to account for both constant and tertiary creep strains, Sarabi [10] introduced a time-dependent viscosity *η*_2_, Equation (2).

Both the Findley power law model [7], Equation (3), and the Findley modified power law model [11], Equation (4), are empirical power functions describing creep with an elastic strain and a transient, non-elastic strain. Equation (3) was employed to describe creep for a variety of materials, such as polymers [12,13,14], metals [7], and concrete [15]. However, some materials show a non-linear viscoelastic behavior in cases of higher initial stresses. Thus, Equation (4) provided successful estimates of long-term creep on the basis of short-term creep data at higher stress levels [11].

The models of Burgers and Findley are widely used to depict the creep behavior of polymers and polymer composites. In Table 1, further models are listed (Equations (5)–(7)) using exponential and power functions or power series. These models may provide suitable and better adjustments to measured creep data, as can be found in [16,17,18].

All models of Table 1 allow for fitting adaption of measured creep data. As a result, one can tabulate model parameters with respect to initial stresses and temperatures. An interface adhesion of polymer composites is a hidden parameter within other parameters. It means that the effect of the reduced and time-dependent interface adhesion on the creep behavior of polymer composites has not been considered yet. The aim of this study is to derive a time-dependent compliance function containing the adhesion term on the base of a *cube in cube* model to predict the creep behavior of reinforced polymers.

## 2. Results and Discussion

### 2.1. Measured Creep Compliances and Their Fitting Using Burgers and Findley Models

**Figure 1:**
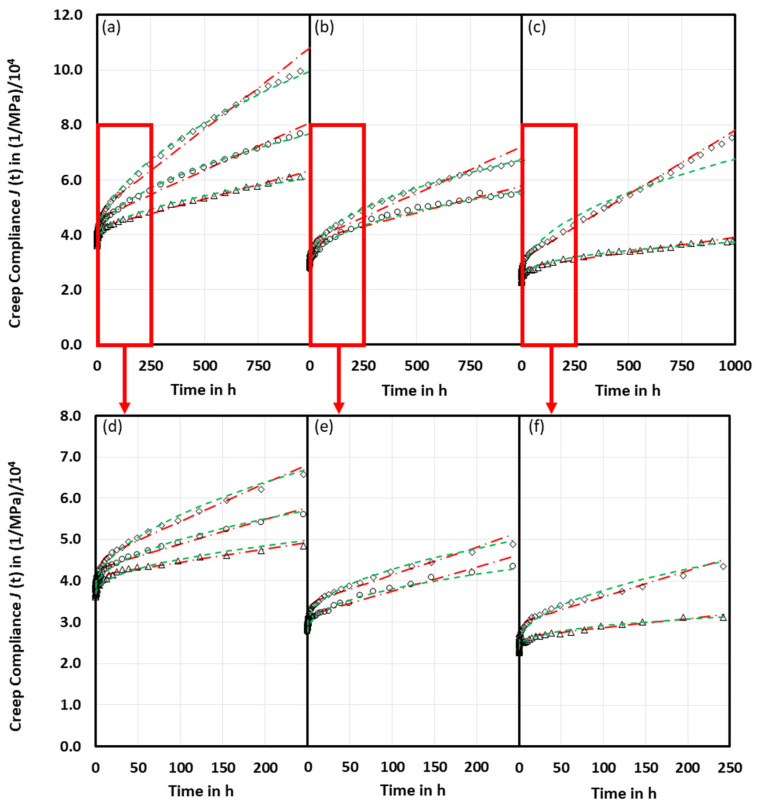
Measured creep compliances (symbols) of neat PBT (**a**,**d**), PBT GB20 (**b**,**e**), and PBT GB30 (**c**,**f**) fitted with Burgers model (dash-point line) and Findley power law model (dashed line) with initial stresses *σ*_0_ = 11 MPa (**∆**), 17 MPa (**o**), and 22 (**◊**) MPa; time intervals of fitting were 1000 h (**a**–**c**) and 250 h (**d**–**f**).

The measured creep compliance curves exhibit an initial elastic compliance followed by primary and partly secondary creep behavior within the measuring time of 1000 h; Figure 1. As expected, the creep compliances increase with time and initial stress and decrease with filler volume content. In the secondary creep range, the creep rates decrease as glass beads are a factor 20 stiffer than the poly(butylene terephthalate) (PBT) matrix, thus, exhibiting negligible creep for small initial stresses [19]. Only PBT GB30 at *σ*_0_ = 22 MPa clearly reached the range of the secondary creep with the constant creep rate.

Burgers model and Findley power law model were used to fit the creep curves; Figure 1. They provide good fits up to 250 h, especially as long as the initial stresses remain small. The determined fitting parameters are summarized in Table 2 and Table 3. In general, the Findley power law model performs better in the investigated time ranging from 250 to 1000 h, respectively. From the creep curves, it is obvious that the secondary creep has not been reached yet, Figure 1a,b,d–f. Except for PBT GB30 at 40% *σ*_max_, the Burgers model performed better as it shows nice linear creep, which can be attributed to a constant slippage rate of polymer chains in load direction [20], Figure 1c.

Fitting the measured creep compliances using the Burgers model shows that the model parameters *E*_1_, *E*_2_, *η*_1,_ and *η*_2_ decrease with initial stresses (=softening effect due to more free volume) and increase with glass beads volume contents (=stiffening effect due to hard fillers), Table 2. In general, the values of the model parameters decrease if the upper time limit is increased from 250 to 1000 h. This behavior is an effect of fitting procedure as viscous flow behavior mainly addressed by a dashpot *η*_1_ comes more into play for longer time ranges with a consequence of decreasing viscosities *η*_1_.

The parameter *E*_1_ can be associated with Young’s modulus of the material, and the *E*_1_ values in Table 3 and Table 4 are similar to Young’s moduli of PBT determined by tensile tests [21]. The first data point of the creep experiment is not determined at the beginning but with a delay of 10 to 20 s. This means that it contains already small creep portions with the consequence that the start value of *E*_1_ can be chosen up to 5% smaller as *E*_2_ may partly compensate for that in the fitting process.

There have been some trials to connect the model parameters with physical properties, e.g., *E*_2_ was associated with the stiffness of amorphous polymer chains [22], which plays a role in the short-time creep stage. However, the moduli *E*_2_ of Table 2 are far too large for amorphous polymers. Similarly, viscosities *η*_1_ and *η*_2_ were connected to damage of crystalline or oriented non-crystalline regions and characteristic times of damaging processes [23]. Nevertheless, as damage mechanisms in crystalline regions differ from those of amorphous regions with respect to physical processes and characteristic times, it is clear that all model parameters except *E*_1_ are only fit parameters.

Fitting of the measured creep compliance curves using the Findley power law model provides values for the model parameters, Table 3, for the upper fitting time limits of 250 h and 1000 h. The parameter *ε*_0_ represents the initial elastic strain, and as expected, it increases with the initial stress and decreases with the content of glass beads. The conversion to the initial modulus *E*_1_ shows coincidence to the moduli determined for the Burgers model and Young’s moduli from the literature within the accuracy of measurements.

The power coefficient *n* ranges from 0.35 to 0.55, meaning that the Findley model can describe only primary creep. As the *n* increases with the initial stresses, it provides enhanced creep. The introduction of glass beads leads to the smaller *n* indicating the reduction of the creep. Interestingly, *n* remains unchanged if the upper fitting time limit is increased from 250 h to 1000 h. This makes the Findley model a good tool for practitioners to predict creep behavior from short-term creep experiments [12,24].

In this evaluation, the determined transient strain *ε*^+^ corresponds to the creep strain after 1 h, which should increase with the initial stress. This is only true for the neat PBT, whereas it decreases for the glass-beads filled PBT. Furthermore, the transient strains *ε*^+^ of the glass bead-filled PBT exceed those of the neat PBT by a factor 2 to 3. This may mean that the glass bead-filled PBT exhibits an enhanced strain rate during the first hour of the creep before the creep equilibrium is established.

The evaluation of the creep compliance curves using empirical models always provides parameter sets depending on the initial stresses and materials compositions (e.g., the content of glass beads). It means that for calculation, one has to measure the creep compliances first with subsequent parameter evaluation before one is able to predict the creep behavior beyond the measured time or for other filler contents.

In the models of Burgers and Findley, the parameter *E*_1_ represents the elastic initial response on the external load. Therefore, Young’s modulus *E*_1_ can be used as an input parameter that takes into account the filler content already before introducing it to the creep models. This can be achieved by the use of *cube in cube* models with various complexity, which were used for more than 60 years to calculate Young’s moduli of particulate composites as a function of matrix modulus *E_M_*, filler modulus *E_F_* and filler volume content *v_F_* [25].

Young’s modulus *E*_1_ is the input parameter to most creep models. Therefore, it seems reasonable to use models to calculate it before introducing it to the creep models. To do so, *cube in cube* models with various complexity were used for more than 60 years to calculate Young’s moduli of the particulate composites as the function of matrix modulus *E_M_*, filler modulus *E_F_* and filler volume content *v_F_* [25].

### 2.2. Determination of the Time Dependent Compliance Function of Glass Beads Filled Composites

**Figure 2:**
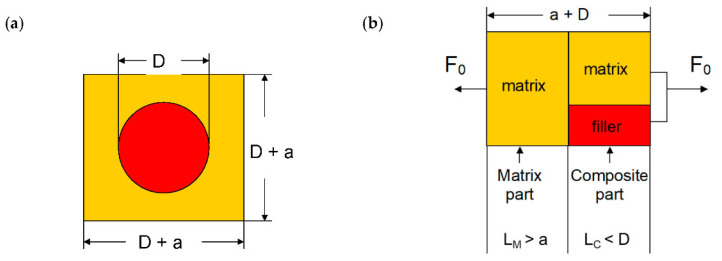
(**a**) Cubic elementary volume (EV) with edge length *D* + *a* containing spherical inclusion of diameter *D*; *a* denotes distance between neighboring inclusions. (**b**) Separation of EV into matrix part and composite part containing now cubic inclusion according to [26] with the external load *F*_0_.

The simplest *cube in cube* models was proposed by Paul [26] and Ishai–Cohen [27] in order to calculate Young’s moduli of particle-filled composites. They assume a cubic elementary volume (EV) with edge length *D* + *a* containing a single inclusion of diameter *D* shown for glass beads in Figure 2a. For modeling purposes, the inclusion has to be transformed into a cube. Then the EV is separated into a matrix part and a composite part according to [26] as it represents the upper bound, Figure 2b. Thus, the length of the matrix part is increased to *L*_M,_ and the length of the composite part is decreased to *L_C_*.

Due to the transformation, *L_C_* becomes (*k D*) and *L_M_* becomes (*a* + 1 − *k*) with the efficiency factor *k*. It considers that less than the maximum cross-section contributes to the stress transfer and thus depends on the particle shapes. For spheres, it is determined by the condition

(8) $$V_{sphere}=\frac{\pi }{6}\;D^{3}=\;k^{3}\;D^{3}=V_{cube}\;\;\;\Rightarrow \;\;\;k=k_{sphere}=\sqrt[{3}]{\frac{\pi }{6}}\;≅0.81$$

It is obvious that the strains of EV, matrix part, and composite part are different. The strain of EV *ε_EV_* represents the macroscopic strain measured in a creep experiment, whereas the strains of matrix part *ε_M_* and composite part *ε_C_* represent “unknown” microscopic strains yet, which are related to each other for perfect interface adhesion according to [28]

(9) $$\varepsilon_{EV}\left(\varepsilon_{M},\varepsilon_{C}\right)=\frac{1-k+d}{1-d}\varepsilon_{M}+\frac{k}{1-d}\;\varepsilon_{C}=\left(1-\frac{k}{1-d}\right)\varepsilon_{M}+\frac{k}{1-d}\;\varepsilon_{C}$$

with the normalized inclusion distance *d* to which a filler volume content *v_F_* is linked by

(10) $$v_{F}={\left(\frac{k}{1-d}\right)}^{3}$$

Thus, the microscopic strains are linked to the macroscopic strain by the dimensional ratio of the EV and the inclusion or by the filler volume content. However, if the cross-sectional ratio of filler inclusion and matrix exceeds 1 (identical cross-sections of filler particle and matrix), the related filler volume content *v_F_* exceeds 26%. This yields increasing shear stresses [28]; hence, the cross-sectional ratio represents a restricting/limiting parameter in this approach. In the creep experiment, the test bars are loaded with a constant force *F*_0_. This allows the expression of the strains in terms of initial stress *σ*_0_ and moduli of EV *E_EV_*, matrix part *E_M_*, and composite part *E_C_*.

(11) $$\mathrm{strain}\;\mathrm{of}\;\mathrm{EV}\;\;\;\varepsilon_{EV}=\frac{\sigma_{0}}{E_{EV}}$$

(12) $$\mathrm{strain}\;\mathrm{of}\;\mathrm{matrix}\;\mathrm{part}\;\;\;\varepsilon_{M}=\frac{\sigma_{0}}{E_{M}}$$

(13) $$\mathrm{strain}\;\mathrm{of}\;\mathrm{composite}\;\mathrm{part}\;\;\;\varepsilon_{EV}=\frac{\sigma_{0}}{E_{F}\;\left(\frac{k^{2}}{{\left(1+d\right)}^{2}}\right)+E_{M}\;\left(1-\frac{k^{2}}{{\left(1+d\right)}^{2}}\right)}$$

Introduction of Equations (11)–(13) to Equation (9) yields

(14) $$\frac{\sigma_{0}}{E_{EV}}=\left(1-\frac{k}{1-d}\right)\frac{\sigma_{0}}{E_{M}}+\frac{k}{1-d}\;\frac{\sigma_{0}}{E_{F}\;\left(\frac{k^{2}}{{\left(1+d\right)}^{2}}\right)+E_{M}\;\left(1-\frac{k^{2}}{{\left(1+d\right)}^{2}}\right)}$$

During creep experiments, there may occur delamination between the matrix and filler depending on time and initial stress. This reduces the interface adhesion and the load transfer to the filler particles. The structure of Equation (14) allows for the introduction of an adhesion factor *k_adh_*, or an adhesion function *k_adh_*(*t*) in case of a time dependency. The square of the adhesion function can be geometrically interpreted as the effectively available cross-section for stress transfer [28]. Thus, it is possible to adjust it between *k_adh_* = 0 (no adhesion) and *k_adh_* = 1 (perfect adhesion). After dividing Equation (14) by the initial stress *σ*_0_, one ends up with the time-dependent creep compliance of the EV

(15) $$J_{EV}\left(t,\sigma_{0}\right)=\frac{1}{E_{EV}\left(t,\sigma_{0}\right)}=\left(1-\frac{k}{1-d}\right)\;J_{M}\left(t,\sigma_{0}\right)+\underset{=constant\;for\;\sigma_{0}\ll \;\sigma_{max}}{\underset{︸}{\frac{k}{1-d}\;\frac{1}{k_{adh}^{2}\left(t\right)\;\;E_{F}\;\left(\frac{k^{2}}{{\left(1+d\right)}^{2}}\right)+E_{M}\;\left(1-\frac{k^{2}}{{\left(1+d\right)}^{2}}\right)}}}$$

The creep of the composites part can be considered elastic because the external load acts mainly on the filler as it is distributed between filler and matrix by the ratio *E_F_*:*E_M_*. This means that the matrix of the composites part experiences much less stress than the matrix part. Therefore, its viscoelastic behavior can be neglected here.

Equation (15) follows that the time dependency of the composite creep *J_EV_*(*t,σ*_0_) is completely determined by the matrix creep *J_M_*(*t,σ*_0_) for the same given initial stress *σ*_0_ and that for not too high initial stresses, the composite part contributes to the creep of particle-filled composite only elastically representing the time-independent constant term. Furthermore, quantitative information about the time-dependent interface adhesion is accessible via creep experiments as all parameters of Equation (15) are known except for *k_adh_*.

**Figure 3:**
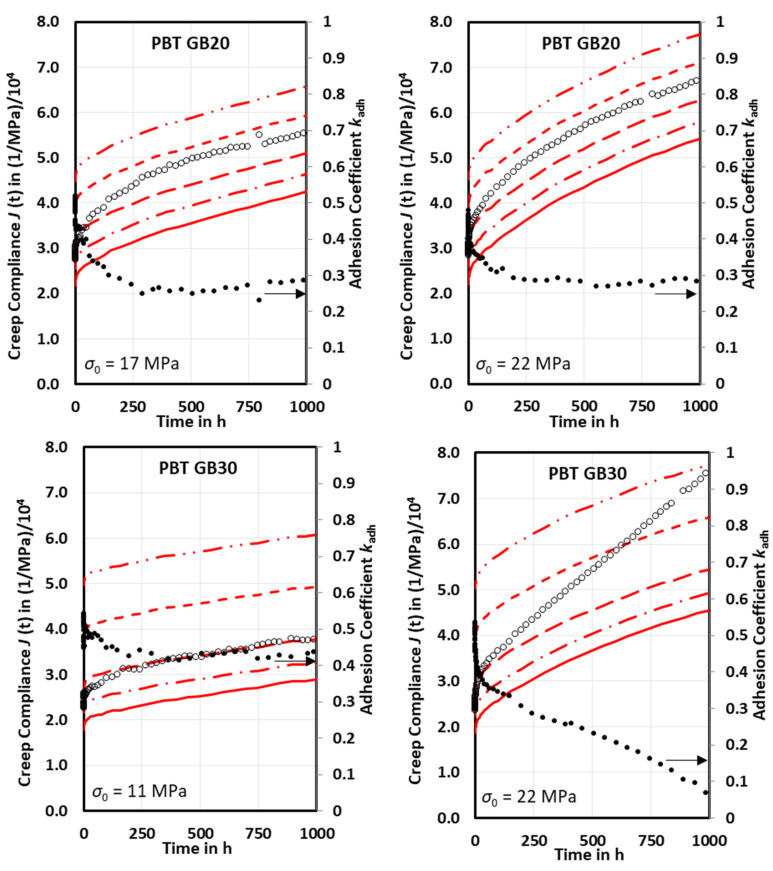
Comparison of measured creep compliances (o) of PBT GB20 and PBT GB30 to calculated creep compliances with adhesion coefficients *k_adh_* = 0, 0.2, 0.4, 0.6, and 1 (lines from top to bottom) for various initial stresses; point by point evaluated adhesion coefficients (●) exhibit clearly time dependency.

If the measured creep compliance curves of neat PBT are introduced in Equation (15), creep compliance curves of particle-filled composites can be calculated for any given adhesion factor. The calculated creep compliance curves for PBT GB20 and PBT GB30 are shown in Figure 3 as the lines for the adhesion factors 0, 0.2, 0.4, 0.6, and 1.

The adhesion factor *k_adh_* = 1 leads to the lowest creep compliance curves (solid lines), with values being smaller than measured creep compliance curves, Figure 3. With the lower adhesion factors, the calculated creep compliances achieve coincidence with the measured ones. Furthermore, the slope of the measured creep compliance curves exceeds the slope of the calculated ones. This means that the adhesion factor decreases with time. A pointwise evaluation provides the time-dependent adhesion function, Figure 3. Over 250 h, the adhesion factors *k_adh_* decrease from 0.55 to 0.36 (PBT GB20 at *σ*_0_ = 17 MPa), 0.50 to 0.32 (PBT GB20 at *σ*_0_ = 22 MPa), 0.53 to 0.45 (PBT GB30 at *σ*_0_ = 11 MPa), and 0.57 to 0.34 (PBT GB30 at *σ*_0_ = 22 MPa) and it decreases faster at the higher initial stresses. Between 250 and 1000 h, the adhesion factors tend towards an asymptotic final adhesion factor except for PBT GB30 at *σ*_0_ = 22 MPa, which decreases linearly due to the secondary creep. The adhesion factors were found to decrease logarithmically with time. Therefore, the time-dependent adhesion factors were fitted using the function

(16) $$k_{adh}\left(t\right)=-\Delta k_{adh}*\ln\left(t\right)+k_{adh}^{t=1\;h}$$

with the incremental slope $\Delta k_{adh}$ and initial adhesion factor $k_{adh}^{t=1\;h}$. The fit parameters are depicted in Table 4. For the fitting time limit of 250 h, both $\Delta k_{adh}$ and $k_{adh}^{t=1\;h}$ increase with the initial stress. For 1000 h the $k_{adh}^{t=1\;h}$ also increases with initial stress. However, for the $\Delta k_{adh}$ the decrease is found for PBT GB20 and the increase for PBT GB30, which can be attributed to the effects of the asymptotic behavior for long times. For PBT GB30 at 22 MPa, the segmental fitting procedure due to the occurrence of the secondary creep provides better fitting of the measured creep compliance.

**Figure 4:**
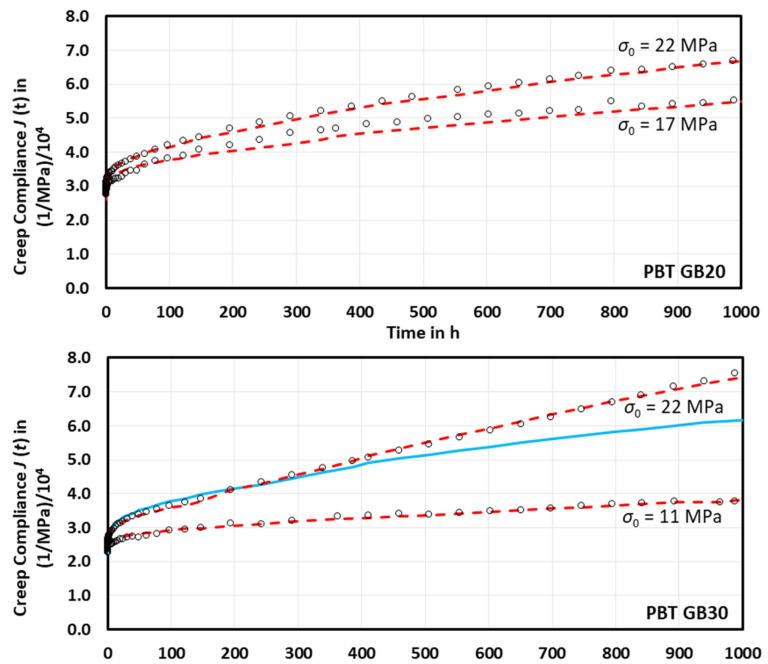
Comparison of initial stress-dependent measured creep compliances (o) of PBT GB20 and PBT GB30 to calculated creep compliances (dashed lines) using Equations (15) and (16); full line represents the extrapolation of 250 h fit.

The introduction of Equation (16) to Equation (15) allows for good adjustment of the measured creep compliances, Figure 4. Only PBT GB30 at 22 MPa does not allow adequate fitting in the long-term creep range from 200 to 1000 h. Due to the linear decrease of *k_adh_*(*t*) at times exceeding 150 h, the fitting was performed in two steps: firstly, with Equation (16) between 0 and 150 h, and secondly, with a corresponding linear approach between 150 and 1000 h.

## 3. Materials and Methods

### 3.1. Materials

Injection molded test bars (type 1A according to ISO 527-2) were manufactured using poly(butylene terephthalate) as neat material (PBT–Ultradur B 2550) and as a matrix for glass bead-filled composites (PBT GB20, Ultradur B4300 K4, and PBT GB30, Ultradur B4300 K6) (BASF SE, Ludwigshafen, Germany). Mechanical properties in terms of Young´s modulus *E*, tensile strength *σ*_m_, and elongation at tensile strength *ε*_m_ are shown in Table 5.

### 3.2. Methods

#### 3.2.1. Creep Tests

Creep tests were performed according to DIN EN ISO 899-1 using a Zwick 1411 creep stand (ZwickRoell, Ulm, Germany – software testXpert) equipped with optical strain measurements at 23 °C and 50% r.h. [29]. The initial stresses *σ*_0_ were chosen to be 11, 17, and 22 MPa, respectively, for neat PBT. This corresponds roughly to yield strength ratios of 20, 30, and 40%. Initial stresses *σ*_0_ were chosen to be 17 and 22 MPa for PBT GB 20 and 11 and 22 MPa for PBT 30 GB. Creep strains were measured for maximum of 1000 h. Subsequently, the creep strain curves were corrected for errors in the measured initial length due to non-vertical mounting and sliding in clamps using a method proposed in [30].

#### 3.2.2. Creep Modeling and Determination of Adhesion Factors

Measured creep strain curves were converted to creep compliance curves and, in the first step, fitted with Burgers model, Equation (1), and Findley power law model, Equation (3), using an Excel solver in the least square approximations for 250 and 1000 h. In the second step, the model parameters were adjusted manually using creep compliance curves in a time logarithmic scale to compensate for the domination of the short-term range. The extended *cube in cube* model, Equation (15), was used to calculate the creep compliances *J_EV_*(*t, σ_0_, v_F_*) of the glass bead-filled PBT composites in a pointwise manner to determine time-dependent interfacial adhesion factors. If the matrix creep compliance has been determined experimentally, all quantities in Equation (15) are known except for *k_adh_*(*t*). Thus, Equation (16) is used to determine the adhesion functions *k_adh_*(*t*) by fitting the time-dependent adhesion factors using Excel solver and then introduced to Equation (15).

## 4. Conclusions

It is known that the creep behavior of composites depends on filler/fiber volume content, initial stress as well as delamination between filler and matrix occurring at long-lasting or high loads. The creep behavior was investigated on poly(butylene terephthalate) (PBT), and its glass beads reinforced composites. First, the measured creep compliance curves were evaluated for 250 and 1000 h using the empirical models of Burgers and Findley. It turned out that the Findley model performs better as long as there is only the primary creep. If secondary creep occurs, the Burgers model is more suitable. The values of the model parameters show that increasing filler volume content leads to stiffening and increasing initial stress to softening. For predicting purposes, the problem is that one gets different parameter sets for each initial stress, and changes in interface adhesion are hidden within them. Both models contain Young’s modulus *E*_1_ as a parameter. Therefore, Paul’s *cube in cube* model, being an elementary volume approach (EV), was used as it offers the chance to introduce *E*_1_ as a filler volume-dependent quantity. Further, the analysis showed that the separation of the EV into the matrix and composite parts leads to time-dependent compliance, which only depends on the creep compliance of the matrix and the mean distance between filler particles or filler volume content. In the composite part, the majority of the stress is transferred to the stiff filler particles. Thus, it contributes only with the constant value to the creep compliance as long as the initial stresses remain small with respect to the tensile strengths. However, the creep compliance of the composites part has to include interface adhesion properties—namely, perfect adhesion. Due to its mathematical structure, it allows for the introduction of the adhesion factor having values between 0 and 1. This enables the elucidation of the time-dependent adhesion factors. This approach offers (design) engineers the chance to predict the realistic creep behavior of particulate composites for any filler volume content if the creep compliance of the matrix for any given initial stress and the adhesion factor is available.

## Figures and Tables

**Table 1:** Some models of time- and stress-dependent creep compliances for polymers.

Models	Creep Strain/Creep Compliance	
Burgers	$J\left(t,\sigma_{0}\right)=\left(\frac{1}{E_{1}}+\frac{1}{E_{2}}\left(1-e^{-\frac{E_{2}}{\eta_{2}}\;t}\right)+\frac{t}{\eta_{1}}\right)$	(1)
	with time *t*, initial stress *σ*_0_, stiffnesses of Burgers model *E*_1_, *E*_2_ and viscosities of Burgers model *η*_1_, *η*_2_	
Modified Burgers	$J\left(t,\sigma_{0}\right)=\left(\frac{1}{E_{1}}+\frac{1}{E_{2}}\left(1-e^{-\frac{t}{\left(a\tau^{b}\right)}}\right)+\frac{t}{\eta_{1}}\right)$	(2)
	with relaxation time *τ* and fitting parameters *a* and *b*	
Findley power law	$J\left(t,\sigma_{0}\right)=\frac{\varepsilon_{0}}{\sigma_{0}}+\frac{\varepsilon^{+}}{\sigma_{0}}t^{n}=\frac{1}{E_{1}}+\frac{\varepsilon^{+}}{\sigma_{0}}{\left(\frac{t}{t_{1}}\right)}^{n}$	(3)
	with initial strain *ε*_0_, transient strain *ε*^+^, exponent *n*, reference time *t*_1_	
Findley modified power law	$J\left(t,\sigma_{0}\right)=\frac{\varepsilon_{0}}{\sigma_{0}}\sinh\left(\frac{\sigma_{0}}{\sigma_{1}}\right)+\frac{\varepsilon^{+}}{\sigma_{0}}t^{n}\sinh\left(\frac{\sigma_{0}}{\sigma_{1}}\right)$	(4)
	with reference stress *σ*_1_	
Bailey–Norton	$J\left(t,\sigma_{0}\right)=A\;\sigma_{0}^{m-1}\;{\left(\frac{t}{t_{1}}\right)}^{n^{+}}$	(5)
	with coefficient *A* and exponents *m* and *n*^+^	
Power series	$J\left(t,\;\sigma_{0}\right)=J_{0}+\sum_{i=1}^{m}J_{i}{\left(\frac{t}{t’}\right)}^{i}$	(6)
	with reference time *t*’, relaxation strengths *J*_i_ and index *i*	
Prony–Dirichlet series	$J\left(t,\;\sigma_{0}\right)\;=J_{0}+\sum_{i=1}^{m}J_{i}\left(1-e^{\left(-\frac{t}{\tau_{i}}\right)}\right)$	(7)
	with relaxation times *τ_i_*	
	with relaxation times *τ_i_*	

**Table 2:** Initial stress-dependent parameters of Burgers model according to fitting creep compliance of neat PBT, PBT GB20, and PBT GB30 showing effect of chosen fitting time ranges.

Range of Fitting Time	GB Content	Initial Stress *σ*_0_	Parameters of Burgers Model			
			*E*_1_	*E*_2_	*η*_1_	*η*_2_
h	w%	MPa	MPa		GPa h	
0 to 250 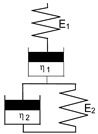	0	11	2720	21,900	3170	115
		17	2620	21,340	1680	92
		22	2570	15,900	1090	74
	20	17	3540	28,100	1750	108
		22	3380	18,890	1530	66
	30	11	4220	35,940	4490	174
		22	4080	18,770	1590	47
0 to 1000	0	11	2710	19,160	4480	219
		17	2600	14,160	2840	141
		22	2560	10,684	1660	128
	20	17	3500	24,100	3310	123
		22	3340	18,500	2370	86
	30	11	4180	23,740	9110	384
		22	4060	15,910	2120	64

**Table 3:** Initial stress-dependent parameters of Findley power law model according to fitting creep compliance of neat PBT, PBT GB20, and PBT GB30 showing effect of chosen fitting time ranges.

Range of Fitting Time	GB Content	Initial Stress *σ*_0_	Parameters of Findley Power Law Model			
			*ε*_0_	*ε* ^+^	*n*	*E*_1_ = *σ*_0_/*ε*_0_
h	w%	MPa	10^−2^	10^−6^	-	MPa
0 to 250	0	11	0.41	2.21	0.47	2690
		17	0.64	2.54	0.52	2657
		22	0.86	3.40	0.55	2545
	20	17	0.47	8.73	0.42	3629
		22	0.62	8.36	0.46	3529
	30	11	0.27	7.58	0.35	4149
		22	0.56	6.23	0.48	3907
0 to 1000	0	11	0.41	2.23	0.47	2666
		17	0.64	2.54	0.52	2657
		22	0.86	3.41	0.55	2546
	20	17	0.47	9.06	0.42	3636
		22	0.62	8.36	0.46	3526
	30	11	0.26	8.02	0.35	4165
		22	0.56	6.63	0.48	3929

**Table 4:** Fit parameters $k_{adh}^{t=1\;h}$ and $\Delta k_{adh}$ of time-dependent adhesion factors for fitting times ranging from 0 to 250 h and 0 to 1000 h. R^2^ shows the correlation coefficient of measured and fitted curves.

Range of Fitting Time	GB Content	Initial Stress	Fit Parameter of Time-Dependent Adhesion Factor		
		*σ*_0_	[Δ*k*]_*adh*	$k_{adh}^{t=1\;h}$	R_2_
h	w%	MPa	-	-	%
0 to 250	20	17	0.017	0.45	95.2
		22	0.016	0.41	99.4
	30	11	0.008	0.49	92.4
		22	0.021	0.45	99.0
0 to 1000	20	17	0.022	0.45	94.6
		22	0.017	0.41	99.3
	30	11	0.009	0.49	92.5
		22	0.027	0.44	97.9
ln		0 < t < 150 h	0.020	0.45	99.4
lin		150 h < t < 1000 h	0.123	0.97	99.6

**Table 5:** Mechanical properties of PBT composites determined according to ISO 527.

Weight Content of Glass Bead	Volume Content of Glass Bead	Young’s Modulus	Tensile Strength	Elongation at Tensile Strength
*v_F_*	*v_F_*	*E*	*σ*_max_	*ε*_max_
-	-	MPa	MPa	%
0	0	2754 ± 25	59 ± 0.1	10.8 ± 1.4
20	0.12	3691 ± 33	57 ± 0.1	3.2 ± 0.2
30	0.19	4408 ± 39	55 ± 0.1	2.0 ± 0.6

## Data Availability

The data obtained and analyzed during the research can be found in ZENODO open repository maintained by CERN https://doi.org/10.5281/zenodo.7284165 (accessed on 18 October 2022).
